# Patients’ Identification, Management and Prevention of Adverse Drug Reactions: A Cross-Sectional Survey of Patients with Severe Adverse Drug Reactions

**DOI:** 10.3390/jcm13144165

**Published:** 2024-07-16

**Authors:** Warisara Srisuriyachanchai, Anthony R. Cox, Narumol Jarernsiripornkul

**Affiliations:** 1 Division of Clinical Pharmacy, Faculty of Pharmaceutical Sciences, Khon Kaen University, Khon Kaen 40002, Thailand; warisara.sr@kkumail.com; 2 Department of Pharmacy, School of Health Sciences, College of Medicine and Health, University of Birmingham, Birmingham B15 2TT, UK

**Keywords:** adverse drug reaction, identification, management, prevention, severity

## Abstract

**Background:** Few studies have been conducted on how patients identify, manage, and prevent severe adverse drug reactions (ADRs). This study aimed to explore the reasoning that patients use to identify symptoms of severe ADRs and the methods they employ to manage and prevent them. **Methods:** A cross-sectional survey using structured telephone interviews was administered to patients with a self-assessed severe ADR and to patients with serious skin ADRs from a hospital medical record database (in-patient and out-patient) from 1 September 2016 to 1 September 2019. Patients identified via the medical records were asked to assess their ADR for severity, and only patients that rated their ADR as severe were followed up with a telephone interview. Structured telephone interviews were conducted with respondents by a research pharmacist and audio-recorded. **Results:** A total of 722 patients with a severe ADR were identified, with 300 completing the interview (41.6%). The most frequently cited reasons for classifying ADRs as severe was worsening ADR symptoms (58.3%), severe ADR symptoms (44.4%), and ADR symptoms interfering with their life (36.4%). Only severe ADR symptoms were significantly different between the questionnaire and the medical records database groups (*p* = 0.007). The most frequent method of ADR management was discontinuation of drug by physicians (88.3%). About 79.0% of patients stated that they increased their carefulness when using other drugs after experiencing ADRs. The main method patients used to prevent ADRs was informing healthcare professionals (HCPs) about their drug allergy history (65.7%). **Conclusions:** Worsening ADR symptoms were often used to identify severe ADRs. However, HCPs were mainly responsible for the management and prevention of severe ADRs. Increasing awareness of ADRs by HCPs, and providing additional drug information, may improve patient safety.

## 1. Introduction

Adverse drug reactions (ADRs) are a major public health concern, with one study estimating that fatal ADRs were between the fourth and sixth leading cause of death in the United States [1]. This early meta-analysis of ADR studies determined that the overall incidence of serious ADRs was 6.7% [1], with later studies showing that 7% to 22% of reported ADRs could be classified as severe [2,3]. Severe ADRs can be life-threatening, cause disabilities, and result in prolonged hospital stays, hospitalization, or the need for intensive medical care. A previous study showed that ADRs led to 9.1% of patients requiring intensive medical care, 0.7% experiencing permanent harm, and 0.9% suffering directly or indirectly fatal outcomes [4]. This is also consistent with a previous study that found 0.1% of severe ADRs resulted in death [5]. Previous studies on the severity levels of ADRs have shown that patients aged 30–50 years, receiving concomitant drugs, experiencing disturbances in daily life, and having higher levels of bothersomeness and anxiety about their ADR reported higher ADR severity levels [3,6,7,8]. The skin and appendages were the most commonly affected organ systems, accounting for 52.25% of cases, and antimicrobials were the major causative drug class, responsible for 40.28% of cases [9]. This is also in line with a previous study from India, which found that the most common causative drug class was antibiotics (47.58%) [10]. The severity levels of ADRs and concerns about their symptoms can influence patients to self-report [11,12].

Spontaneous reporting is a method of ADR identification that relies on healthcare professionals (HCPs) to identify and report suspected ADRs. The major limitation of this method is under-reporting [13]. Patient reporting of ADRs aims to increase the number of reported ADRs and is, therefore, another way to improve treatment safety [14]. Patients provide more extensive details about their reactions in their reports than those from HCPs, and they more often describe the effects of ADRs on their lives [15,16]. HCPs tend to focus on objective data (e.g., start date of ADR) and more clinically relevant information whereas patients provided more subjective data (e.g., the personal impact of the ADR) [16]. The early detection, evaluation, and monitoring of ADRs can reduce harm to patients and improve their quality of life.

Several previous studies have developed and tested ADR causality assessment tools for patients [17,18,19], but there has been little analysis of the methods or reasons for identifying or assessing severe ADRs. One previous study conducted telephone interviews to explore how patients identify and assess symptoms as ADRs. The results showed that the temporal relationship between the onset of symptoms and the taking of the drug was the most frequently mentioned factor in assessing for a possible ADR [20], confirming the findings of other studies [21,22]. As most studies have only focused on ADR reporting [21,22,23,24,25], with little attention paid to the details of severe ADR identification, management, and prevention by patients, a better understanding of the methods employed by patients for identifying, managing, and preventing severe ADRs could benefit the ADR monitoring process and improve medication safety.

To date, there have been limited studies conducted in Thailand on how patients identify, manage, and prevent severe ADRs. Such understanding could help to improve the rate of patient ADR reporting. Therefore, this study aimed to explore the reasoning patients use to identify severe ADRs and the methods they use to manage and prevent them.

## 2. Materials and Methods

This study is a cross-sectional study conducted at two university hospitals in Northeast Thailand (Srinagarind Hospital and Queen Sirikit Heart Center of the Northeast) from December 2020 to February 2021. Eligible participants were patients who rated their ADRs as severe by Numerical Rating Scale (NRS) with a score of 7–10 in a self-assessed questionnaire administered in our previous study [8] and outpatients and inpatients who had experienced a serious skin ADR over a period of 3 years (1 September 2016 to 1 September 2019) according to the medical records database at Srinagarind Hospital in Khon Kaen province. Eligible participants were asked to rate their ADR severity levels before starting the telephone interview. Only patients who self-assessed their ADR as severe in a pre-interview were followed up with a telephone interview. The study excluded patients who could not communicate in the Thai language, patients who were unable to be contacted, and patients who refused to participate.

The factors collected from the hospital database were patient age, gender, number of underlying diseases, and number of concomitant drugs. Serious skin reactions were identified using the International Classification of Disease and Related Health Problem 10th Revision (ICD-10) and retrieved from the hospital database. The eight ICD-10 categories used were as follows: L27.0 (Generalized skin eruption due to drugs and medicaments for DRESS), L27.1 (Fixed drug eruption), L50.0 (Urticaria), L51.1 (Stevens–Johnson syndrome; SJS), L51.2 (Toxic epidermal necrolysis; TEN), L53.8 (Acute generalized exanthematous pustulosis; AGEP), T78.2 (Anaphylaxis), and T78.3 (Angioedema). The structured interview guideline was developed by two researchers based on previous studies [3,20,26]. It contained four main topics: (i) detail of ADR experiences; (ii) method of severe ADR identification; (iii) method of severe ADR management; (iv) method of recurrent drug allergy prevention. The structured interview was validated by three experts (one physician, two clinical pharmacists) using an index of item objective congruence (IOC) technique. All questions passed the content validity with IOC > 0.5 for each item. All three experts were also asked to consider the questionnaire language and flow for ease of understanding. The final structured interview was re-adjusted following suggestions from the validation test. For respondents who were unable to provide information, their parents or care-giver were interviewed. All interviews were conducted by a research pharmacist by telephone interview in a private room. Patient interviews took 10–15 min and audio recordings were collected for each interview. All questions were asked based on the interview guideline. Open questions were used to ask patients to explain their thoughts or reasoning. All data were collected between December 2020 and February 2021.

The validated structured telephone interviews were analyzed using SPSS for Windows version 26.0. Demographic data, frequency, and type of ADRs were analyzed using descriptive statistics. Pearson’s chi-squared and Fisher’s exact test were used to compare subgroups for categorical data, and independent-sample *t*-tests were used for continuous data. The Mann–Whitney U test was used to determine differences in continuous data for non-parametric variables. The results with *p*-values less than 0.05 were considered statistically significant. The interview transcripts were analyzed manually by a research pharmacist. The research pharmacist first listened to the interviews and noted the main keywords, then the data were categorized into major sentences. The study was approved by the Ethics Committee of the Khon Kaen University Ethics Committee for Human Research (Number HE621444 on 16 December 2019).

## 3. Results

### 3.1. Response Rate

There were 2314 patients included in the study, made up of patients from the previous study [25] and patients who had experienced serious skin ADRs over 3 years (1 September 2016 to 1 September 2019) according to the medical records database at Srinagarind Hospital in Khon Kaen province. A total of 1800 patients (77.8%) had provided their telephone number for contact and 722 of these patients (40.1%) had a history of a severe ADR. Four hundred and twenty-two patients (58.4%) could not be contacted or refused to participate in the study and structured interviews were undertaken with 300 patients between the 1 December 2020 and the 28 February 2021.

### 3.2. Demographic Data

Demographic details of the study respondents are shown in Table 1. The majority of respondents were female (69.3%) and the average age was 46.0 ± 19.81 years (min–max 1–88). Half of the patients were aged less than 50 years (52.0%). Almost three-quarters of respondents (73.7%) claimed that they had less than one comorbidity and more than half of respondents (58.3%) claimed that they had used less than three medicines. The mean duration of the interviews was 13.3 ± 3.31 min (min–max 5.2–25.0).

### 3.3. Severe ADR Identification

The reasons respondents said that they used to confirm an association between their symptoms and a suspected drug were shown in Table 2. The most frequently used reason was that the symptoms occurred after the drug was administered (33.3%), followed by no co-medications were administered during their suspected ADR (18.0%), and thirdly that ADR symptoms reappeared when the drug was re-administered (6.0%). There were no significant differences between the questionnaire and the medical records database groups. About one-third of respondents (32.0%) did not think that their unusual symptoms were caused by medicines.

The respondents were asked questions about how they decided if their ADRs were severe. A total of 300 respondents, 187 (62.3%) stated their ADRs as severe. Among the respondents, the top three reasons for identification of an ADR as severe were ADR symptoms were getting worse (58.3%), ADR symptoms were characterized as severe (44.4%), and ADR symptoms interfered with their life (36.4%). The results were similar in both the questionnaire and the medical records database groups. The only characteristic that was significantly different between the questionnaire and the medical records database groups (*p* = 0.007) was for ADR symptoms characterized as severe (Table 2).

### 3.4. Severe ADR Management

#### 3.4.1. ADR Management

The most frequently used method for management of severe ADRs was discontinuation of the suspected drug (98.3%). More patients in the questionnaire group made the decision to discontinue the suspected drug on their own than in the medical records database group (46.7% vs. 28.8%, respectively, *p* = 0.003). Patients in the medical records database group relied upon the advice of other people to discontinue the suspected drug more frequently than in the questionnaire group (95.7% vs. 81.5%, respectively, *p* < 0.001). For both groups, most of the patients (88.3%) discontinued the suspected drug based on their physicians’ recommendation. Overall, 39 (13.0%) of respondents continued to take the suspected drug, with more patients in the questionnaire group continuing to take the drug than in the medical records database group (19.6% vs. 10.1%, respectively, *p* = 0.025). The major reason provided by patients for continuing to take the drug was to wait and observe their ADR symptoms (8.3%). Most respondents (98.7%) reported that the dose of their suspected drug was not increased or decreased (Table 3).

Some respondents stated that they continued to take their suspected drugs because they believed that their unusual symptoms were not caused by the medication (25; 8.3%) and that the suspected drugs were essential for their diseases (11; 37.0%). A majority of respondents (284; 94.7%) found that their ADR symptoms disappeared after discontinuing the suspected drugs, while the small number of respondents who continued to take their suspected drugs and did not treat their ADR symptoms (27, 9.0%) reported that their ADR symptoms remained the same (14; 4.7%) or became worse (13; 4.3%). A total of 86.3% of the respondents did not re-challenge with the suspected drug after discontinuing it. Physicians were the main HCPs consulted by the respondents for their ADR symptoms (276; 92.0%).

#### 3.4.2. Carefulness for Using Other Drugs

All respondents were asked about their carefulness when using other drugs after experiencing an ADR. While an overall majority of respondents (79.0%) reported being more cautious when using other drugs after experiencing an ADR, there were significant differences between the questionnaire and the medical records database groups. A much higher proportion of respondents in the questionnaire group reported an increase in their carefulness compared to the medical records database group (90.2% vs. 74.0%, respectively, *p* = 0.002). The most commonly used methods applied by respondents to increase their carefulness were to consult an HCP before commencing the drug (40.9%), followed by observing for any symptoms developing after administration of a drug (33.8%), and reading the drug label (27.0%). There were no significant differences in the methods used to increase carefulness between the questionnaire and the medical records database groups. The internet was the main source of drug information used by patients with severe ADRs (19.8%) (Table 4).

### 3.5. Severe ADR Prevention

The method most frequently used by respondents to prevent a recurrent severe ADR was to inform their HCPs about their severe ADR history (195, 65%). Other commonly used methods included avoiding drugs that had previously caused a severe ADR reaction (58, 19.3%), consulting a physician or pharmacist before taking medication (36, 12.0%), and making sure that a prescribed drug not a drug that has caused a severe ADR (33, 11.0%). Some respondents reported carrying an “Allergy Card” related to a prior ADR and which is issued to patients by HCPs (10.3%). Others reported avoiding the buying and taking of medications without HCP involvement (10.0%). There were no significant differences between the questionnaire and the medical records database groups in the frequency of the methods used (Table 5).

## 4. Discussion

Three hundred patients who had previously experienced an ADR were asked in structured interviews to explain how they identify, manage, and prevent severe ADRs. Patients identified severe ADRs through a worsening of symptoms, especially unusual symptoms such as dyspnea and unconsciousness, which interfered with their daily lives. The most common method of severe ADR management was discontinuation of the suspected drug by physician, and informing HCPs about their drug allergy history was the main method of ADR prevention.

The major strength of our study included the use of structured telephone interviews, which allowed patients to explain their severe ADR experiences in their own words, and the purposive sampling of participants based on important factors for variation. Only patients who had previously rated their ADRs as severe, or patients who had experienced a serious ADR according to the medical records database and self-assessed them as severe, were interviewed. A limitation of the study was the potential for recall bias, as patients were asked to recall past experiences of severe ADRs during their telephone interviews. In the questionnaire group, we asked about all experienced ADRs, not just skin ADRs, which limits our ability to directly compare skin ADRs between groups.

This study found that patients tended to provide more extensive details about the nature of their reactions and the effect of ADRs on their daily lives, confirming the findings of other studies [15,16]. Patients reported that their experience of worsening ADR symptoms was the easiest way to identify severe ADRs, along with the timing relationship between taking a drug and experiencing the symptoms, differences with their experience of their concomitant diseases and response to other medicines, and via re-challenge with the suspected drug. These findings confirmed the reasoning employed by patients to make a report seen in previous studies, including the use of the temporal relationship and the ADR [20,21,22], a lack of co-medication being taken concurrently which might be an alternative explanation, and ADRs recurring after re-challenge with the suspected drug [20]. Reasons not to make a report were also similar, with some patients who did not believe their unusual symptoms were caused by medicines as also seen in prior studies [27].

While most patients in the current study discontinued the suspected drug to manage their ADRs, which is consistent with previous studies [26,27,28], there was a higher proportion of patients discontinuing the suspected drug in the current study [2,26,27]. This could be because the current study only included patients who had experienced severe ADRs, and discontinuation of the suspected drug is more likely in such severe ADRs. The current study also found that after discontinuation of the drug, a higher proportion of respondents reported that they had recovered from their ADR symptoms than in an earlier study [26]. This may be related to the nature of the severe ADRs that were retrieved from the medical records database. Patients did not typically increase or decrease the dose or re-challenge with the suspected drug, in agreement with previous studies [29,30]. This aligns with other studies suggesting that severe adverse events can be managed through dose modification or additional treatment [31,32,33]. The current study also confirmed that physicians were the most common HCP for patient reporting of ADRs [26,34].

Most patients became more careful when using other drugs after experiencing an ADR. Physician consultation before using other drugs was the most frequently used method to increase carefulness. This is in line with previous studies from Thailand in which HCPs were the major sources of information used by patients [26]. Patients also reported increasing observation for unusual symptoms after drug administration and reading drug labels to prevent ADRs, confirming previous studies that found that past ADR experience and drug information leaflets were major sources of information [26]. The ADR prevention methods applied by patients in this study are similar to two recent studies that evaluated patient histories to provide basic knowledge about the importance of allergy cards for enhanced ADR prevention [35,36]. Another study stated that a past history of drug allergy was a factor that might increase the chance of an ADR [37]. Patient histories and information about the suspected drug are important information for ADR management and prevention [38,39]. The current study showed that patients avoided drugs that caused an allergic reaction in the past and sought ADR information from HCPs before using any other medications. The current study also confirmed that most patients required more information about their allergic drugs and agreed that package insert or patient information leaflets and HCPs are beneficial sources of ADR information [40]. In Thailand, patients are more likely to focus on ADR symptoms that affect their daily life. However, HCPs still play an important role and support the ADR monitoring system. Increasing the involvement of patients in ADR reporting does not mean abandoning the promotion of ADR monitoring by HCPs.

## 5. Conclusions

Patients usually tend to report severe ADR symptoms that become worse and affect their daily life. Discontinuation of a suspected drug is the most common patient-reported method of severe ADR management. Consultation with HCPs is the main method of severe ADR prevention. Increasing the awareness of HCPs about ADRs and providing additional drug information may improve severe ADR monitoring and medication safety.

## Figures and Tables

**Table 1:** Demographic characteristics.

Characteristics	Number of Severe ADRs Rated by Patients; *N* (%)			*p*-Value ^a^
	From Questionnaire ^c^ (*n* = 92)	From Database ^d^ (*n* = 208)	Total (*n* = 300)	
**Sex**				
Male	22 (23.9)	70 (33.7)	92 (30.7)	0.092
Female	70 (76.1)	138 (66.3)	208 (69.3)	
**Age (years)**				
≤50	23 (25.0)	133 (63.9)	156 (52.0)	<0.001 *
>50	69 (75.0)	75 (36.1)	144 (48.0)	
Mean ± SD	58.2 ± 14.19	40.7 ± 19.60	46.0 ± 19.81	
Median (range)	58.5 (20–86)	42.5 (1–88)	32.0 (1–88)	<0.001 ^b^*
**Number of comorbidities**				
0–1 disease	56 (60.9)	165 (79.3)	221 (73.7)	0.001 *
≥2 diseases	36 (39.1)	43 (20.7)	79 (26.3)	
**Number of concomitant drugs**				
0–3 items	27 (29.3)	148 (71.2)	175 (58.3)	<0.001 *
≥4 items	65 (70.7)	60 (28.8)	125 (41.7)	

^a^ Pearson’s chi-square test, ^b^ Mann–Whitney U test, * the level of significant different <0.05, ^c^ severe ADRs retrieved from patient questionnaire, ^d^ severe ADRs retrieved from the medical records database.

**Table 2:** Reasons respondents used to describe whether their ADRs were caused by the suspected drug and used to identify severe ADR.

**Reason Used to Identify ADR**	**Number of Severe ADRs Rated by Patients; *N* (%)**			***p*-Value ^a^**
	**From Questionnaire ^c^** **(*n* = 92)**	**From Database ^d^** **(*n* = 208)**	**Total** **(*n* = 300)**	
ADR symptoms occurred after the drug was administered.	37 (40.2)	63 (30.3)	100 (33.3)	0.093
No co-medications were administered during their suspected ADR.	13 (14.1)	41 (19.7)	54 (18.0)	0.246
ADR symptoms reappeared when the drug was re-administered.	4 (4.3)	14 (6.7)	18 (6.0)	0.423
ADR symptoms did not appear after the administration of other drugs.	6 (6.5)	9 (4.3)	15 (5.0)	0.566 ^b^
Symptoms were known to be characteristic of ADRs by the patient.	5 (5.4)	10 (4.8)	15 (5.0)	1.000 ^b^
ADRs had never been like this before.	1 (1.1)	12 (5.8)	13 (4.3)	0.119 ^b^
Change to a new drug after the ADR occurred.	3 (3.3)	3 (1.4)	6 (2.0)	0.375 ^b^
ADR symptoms were different to the symptoms of their underlying diseases.	3 (3.3)	3 (1.4)	6 (2.0)	0.375 ^b^
**Reason Used to Identify Severe ADR**	**Number of Severe ADRs Rated by Patients; *N* (%)**			***p*-Value ^a^**
	**From Questionnaire ^c^** **(*n* = 72)**	**From Database ^d^** **(*n* = 115)**	**Total** **(*n* = 187)**	
Symptoms were getting worse	48 (66.7)	61 (53.0)	109 (58.3)	0.066
Short onset of symptoms after taking medicine	5 (6.9)	5 (4.3)	10 (5.3)	0.512 ^b^
Symptoms were characterized as severe	23 (31.9)	60 (52.2)	83 (44.4)	0.007 *
Symptoms caused hospital admission	5 (6.9)	16 (13.9)	21 (11.2)	0.142
Symptoms interfered with their life	20 (27.8)	48 (41.7)	68 (36.4)	0.053

^a^ Pearson’s chi-square test, ^b^ Fisher’s exact test, * the level of significant different <0.05, ^c^ severe ADRs retrieved from patient questionnaire, ^d^ severe ADRs retrieved from the medical records database.

**Table 3:** Methods used for the management of severe ADRs.

Method	Number of Severe ADRs Rated by Patients; *N* (%)			*p*-Value ^a^
	From Questionnaire ^c^ (*n* = 92)	From Database ^d^ (*n* = 208)	Total (*n* = 300)	
Discontinuation of the suspected drug **	89 (96.7)	206 (99.0)	295 (98.3)	0.328 ^b^
By yourself	43 (46.7)	60 (28.8)	103 (34.3)	0.003 *
By other people	75 (81.5)	199 (95.7)	274 (91.3)	<0.001 *
Physician	72 (78.3)	193 (92.8)	265 (88.3)	0.709 ^b^
Pharmacist	1 (1.1)	2 (1.0)	3 (1.0)	1.000 ^b^
Nurse	2 (2.2)	2 (1.0)	4 (1.3)	0.578 ^b^
Continuation of the suspected drug	18 (19.6)	21 (10.1)	39 (13.0)	0.025 *
Use an additional drug	3 (3.3)	1 (0.5)	4 (1.3)	0.318 ^b^
Adjust suspected drug administration	1 (1.1)	1 (0.5)	2 (0.6)	1.000 ^b^
Wait and observe	12 (13.0)	13 (6.3)	25 (8.3)	0.757
See physician	2 (2.2)	6 (2.9)	8 (2.7)	0.247 ^b^
Dose management				
Dose was not changed	92 (100.0)	204 (98.1)	296 (98.7)	0.316 ^b^
Dose was increased or decreased	0 (0.0)	4 (1.9)	4 (1.3)	0.316 ^b^

^a^ Pearson’s chi-square test, ^b^ Fisher’s exact test, * the level of significant different <0.05, ** respondents could choose more than one answer, ^c^ severe ADRs retrieved from patient questionnaire, ^d^ severe ADRs retrieved from the medical records database.

**Table 4:** Patient carefulness when using other drugs after experiencing an ADR.

Detail	Number of Severe ADRs Rated by Patients; *N* (%)			*p*-Value ^a^
	From Questionnaire ^c^ (*n* = 92)	From Database ^d^ (*n* = 208)	Total (*n* = 300)	
Same carefulness	9 (9.8)	54 (26.0)	63 (21.0)	0.002 *
Increase carefulness	83 (90.2)	154 (74.0)	237 (79.0)	
**Methods applied to increase carefulness**	***n* = 83**	***n* = 154**	***n* = 237**	
- Read drug label	18 (21.7)	46 (29.9)	64 (27.0)	0.176
- Find more information	18 (21.7)	30 (19.5)	48 (20.3)	0.687
- Internet	17 (20.5)	30 (19.5)	47 (19.8)	0.375 ^b^
- Document	0 (0.0)	1 (0.5)	1 (0.3)	1.000 ^b^
- Relatives	1 (1.1)	0 (0.0)	1 (0.3)	0.375 ^b^
- Consult healthcare professionals	29 (34.9)	68 (44.2)	97 (40.9)	0.169
- Observe symptoms after drug administration	26 (31.3)	54 (35.1)	80 (33.8)	0.561

^a^ Pearson’s chi-square test, ^b^ Fisher’s exact test, * the level of significant different <0.05, ^c^ severe ADRs retrieved from patient questionnaire, ^d^ severe ADRs retrieved from the medical records database.

**Table 5:** Methods respondents used to prevent recurrent drug allergy.

Detail	Number of Severe ADRs Rated by Patients; *N* (%)			*p*-Value ^a^
	From Questionnaire ^c^ (*n* = 92)	From Database ^d^ (*n* = 208)	Total (*n* = 300)	
Inform healthcare professionals about their drug allergy history	56 (60.9)	139 (66.8)	195 (65.0)	0.319
Do not use drugs that previously caused an allergic reaction	14 (15.2)	44 (21.2)	58 (19.3)	0.230
Consult a physician or pharmacist before taking medication	13 (14.1)	23 (11.1)	36 (12.0)	0.450
Make sure that a prescribed drug is not an allergic drug	13 (14.1)	20 (9.6)	33 (11.0)	0.249
Carry a drug allergy card	6 (6.5)	25 (12.0)	31 (10.3)	0.149
Do not buy and take drugs by themselves	10 (10.9)	20 (9.6)	30 (10.0)	0.738
Find more information or read drug label	5 (5.4)	17 (8.2)	22 (7.3)	0.402
Cannot be prevented by themselves	6 (6.5)	9 (4.3)	15 (5.0)	0.566 ^b^
Received a warning from friends or relatives about a history of allergic reactions to drugs	1 (1.1)	8 (3.8)	9 (3.0)	0.284 ^b^
Try to stay healthy so they can avoid taking any new drugs	2 (2.2)	6 (2.9)	8 (2.7)	1.000 ^b^
Increased observation of themselves when using drugs	1 (1.1)	5 (2.4)	6 (2.0)	0.671 ^b^
Getting treatment from physicians who had their drug allergy history	4 (4.3)	2 (1.0)	6 (2.0)	0.074 ^b^

^a^ Pearson’s chi-square test, ^b^ Fisher’s exact test, ^c^ severe ADRs retrieved from patient questionnaire, ^d^ severe ADRs retrieved from the medical records database.

## Data Availability

The data presented in this study are available on request from the corresponding author.
